# Prevalence of Physical Frailty and Its Multidimensional Risk Factors in Korean Community-Dwelling Older Adults: Findings from Korean Frailty and Aging Cohort Study

**DOI:** 10.3390/ijerph17217883

**Published:** 2020-10-27

**Authors:** Heeeun Jung, Miji Kim, Yunhwan Lee, Chang Won Won

**Affiliations:** 1 Department of Biomedical Science and Technology, Graduate School, Kyung Hee University, Seoul 02447, Korea; heeeun.jung@khu.ac.kr; 2 Department of Biomedical Science and Technology, College of Medicine, East-West Medical Research Institute, Kyung Hee University, Seoul 02447, Korea; 3 Department of Preventive Medicine and Public Health, Ajou University School of Medicine, Suwon 16499, Korea; yhlee@ajou.ac.kr; 4 Elderly Frailty Research Center, Department of Family Medicine, College of Medicine, Kyung Hee University, Seoul 02447, Korea

**Keywords:** community-dwelling older adults, physical frailty, prevalence, risk factors

## Abstract

Frailty is defined as a state of increased vulnerability to stressors, and it predicts disability and mortality in the older population. This study aimed to investigate the standardized prevalence and multidimensional risk factors associated with frailty among Korean community-dwelling older adults. We analyzed the baseline data of 2907 adults aged 70–84 years (mean age 75.8 ± 3.9 years, 57.8% women) in the Korean Frailty and Aging Cohort Study. The Fried frailty phenotype was used to define frailty. Analyzed data included sociodemographic, physical, physical function, biological, lifestyle, health condition, medical condition, psychological, and social domains. Data were standardized using the national standard population composition ratio based on the Korean Population and Housing Census. The standardized prevalence of frailty and prefrailty was 7.9% (95% confidence interval (CI) 6.8–8.9%) and 47.0% (95% CI, 45.1–48.8%), respectively. The following 14 risk factors were significantly associated with frailty: at risk of malnutrition, sarcopenia, severe mobility limitation, poor social capital, rural dwellers, depressive symptoms, poor self-perceived health, polypharmacy, elevated high-sensitivity C-reactive protein, elevated glycosylated hemoglobin, low 25-hydroxy vitamin D level, longer Timed Up and Go, and low Short Physical Performance Battery score (*p* < 0.05). Physiconutritional, psychological, sociodemographic, and medical factors are strongly associated with frailty.

## 1. Introduction

Frailty is characterized by a significant decline in the functional reserve capacity of multiple organ systems with an increased vulnerability to stressors, leading to a higher risk of adverse health outcomes such as falls, disability, hospitalization, and mortality in older adults [1,2]. The wide range in prevalence among the studies is due to the different definitions of frailty. In a systematic review, the prevalence of frailty in community-dwelling older adults aged ≥65 years was found to vary from 4.0% to 59.1% [3]. The Fried Frailty Phenotype (FFP) and the Frailty Index (FI) represent commonly known as operational definitions of frailty in older adults [3,4]. The physical phenotypic approach, the FFP was defined as the presence of three or more of five physical characteristics: weakness, slowness, weight loss, exhaustion, and low physical activity [1]. Moreover, the FFP was originally constructed from an epidemiological study and has drawn the highest degree of attention of researchers. It has predicted adverse clinical outcomes like mortality. Conversely, many clinical studies have adopted the FI for frailty assessment. The FI, is a comprehensive geriatric assessment composed of a long checklist of clinical conditions and diseases that constitutes the deficit accumulation approach. The FI was composed of psychological, mental, and social as well as physical functions and was expressed as a ratio [4]. In recent studies, FFP has been the most widely used definition of frailty in recent studies [5].

There is a rapid increase in the number of older adults aged ≥65 years globally [6]. According to Statistics Korea, the prevalence of older adults aged ≥65 years in Korea was 14.3% in 2018 and is expected to double by 2028 [7]. Recently, the Korean Longitudinal Study on Health and Aging Study performed in hospital-based populations residing in the city of Seongnam in Korea reported that the prevalence of frailty and prefrailty was 13.2% and 59.4%, respectively [8]. The aging study of the Pyeongchang Rural Area in older adults of Pyeongchang reported the prevalence of frailty and prefrailty as 17.4% and 52.6%, respectively [9]. They identified instrumental activities of daily living (IADL) and activities of daily living (ADL) disability, depression symptoms, dysmobility, malnutrition, incontinence, and medical aid as risk factors for frailty. However, these studies were restricted to a selected residential area in Korea and do not represent community-dwelling older adults.

Frailty is a dynamic reversible state, and identification of the risk factors of frailty will enable prevention and management. Previous studies have identified risk factors for physical frailty, focusing on sociodemographic factors such as age, gender, marital status, education level, and physical factors such as body composition and physical function [10,11,12]. However, more recent studies have identified a wider range of risk and protective factors, including biological, lifestyle, and psychological factors [13]. As risk factors of physical frailty have been identified in multiple domains, it is necessary to comprehensively identify the influential risk factors to prioritize targets.

This study aimed to investigate the age-, sex-, and residence-adjusted prevalence and characteristics of physical frailty in the Korean Frailty and Aging Cohort Study (KFACS). We also identified risk factors with a significant association with physical frailty using multidimensional domains in Korean community-dwelling older adults.

## 2. Materials and Methods

### 2.1. Study Population

The KFACS is a nationwide, longitudinal study, with the baseline survey conducted in 2016–2017 [14]. The KFACS recruited participants using quota sampling methods stratified by sex (male and female in a ratio of 1:1) and age (70–74, 75–79, and 80–84 years in a ratio of 6:5:4, respectively). The participants were recruited from among community-dwelling residents in urban and rural areas in 10 study centers covering different residential locations (urban, suburban, and rural): three from the Seoul Metropolitan Area, two from Gyeonggi Province, and one from each of Gangwon Province, Chungcheongbuk Province, Jeolla-nam Province, Gyeongsang-nam Province, and Jeju Island in South Korea. Of the 3014 participants who were enrolled at 10 centers at baseline, 2907 participants completed the assessment of 5 components of FFP and were selected for the final analysis, after excluding 109 with missing frailty assessment components. The KFACS protocol was approved by the Clinical Research Ethics Committee of Kyung Hee University Hospital (IRB number: 2015-12-103). All participants were given prior explanations and signed consent forms. This study had an IRB approval from the Clinical Research Ethics Committee of Kyung Hee University Hospital (IRB number: 2020-06-062).

### 2.2. Frailty Assessment

Physical frailty was defined using FFP based on weight loss, weakness, slowness, exhaustion, and low physical activity with modified cutoff points [1,14]. Weight loss was defined as a “yes” response to the question “In the last year, have you unintentionally lost more than 4.5 kg?”. Handgrip strength was measured twice for both hands using a hand dynamometer (Takei TKK 5401; Takei Scientific Instruments, Tokyo, Japan). Weakness was defined as a handgrip strength in the lower 20%, adjusted for sex and body mass index (BMI) quartiles based on the KFACS baseline survey. The 4 m usual gait speed was measured using an automatic timer (Gaitspeedometer; Dyphi, Daejeon, Korea), with acceleration and deceleration phases of 1.5 m. Slowness was defined as the lowest 20% of gait speed on the basis of the 4 m usual gait speed stratified by sex and height based on the KFACS population distribution. Exhaustion was defined as a “yes” response to either of “I felt that everything I did was an effort” and “I could not get going” on 3 or more days per week from the Center for Epidemiological Studies-Depression scale. Energy expenditure estimates (kcal/week) for physical activity levels were calculated using the International Physical Activity Questionnaire. Low physical activity was defined as the lowest 20% of sex-specific total energy consumed in a population-based Korean survey of older adults from among the general population (Table S1). Physical frailty scores ranged from 0 to 5. Participants with scores ≥3, 1–2, and 0 were classified as frail, prefrail, and robust, respectively.

### 2.3. Measurements

We obtained information on sociodemographic (age, sex, education level, living status, marital status, residential area, social security benefits, and occupation), lifestyle (smoking status, alcohol consumption, and sleep habits), self-perceived health status, history of falls and hospitalization in the past year, current use of prescription medications, oral health, and self-reported history of medical conditions based on Charlson’s classification [15].

Underweight was defined as a body mass index (BMI) <18.5 kg/m^2^. Appendicular skeletal muscle (ASM) was measured using dual-energy X-ray absorptiometry (DXA) (Lunar, GE Healthcare, Madison, WI, USA and Hologic DXA, Hologic Inc., Bedford MA, USA) or bioelectrical impedance analysis (InBody 72, InBody Co., Ltd., Seoul, Korea, and X-SCAN PLUS II, Jawon Medical Inc., Seoul, Korea). A low ASM mass was defined as the lowest 20% of the KFACS participants. Sarcopenia was defined according to the consensus report of the Asian Working Group for sarcopenia based on low muscle strength, low muscle mass, and/or low physical performance [16]. Low calf circumference was defined as <32 cm [17]. High waist circumference was defined as ≥102 cm for men and ≥88 cm for women [18].

Severe mobility limitation was defined if the patient found it “very difficult” or “impossible” to either walk about 400 m or climb 10 steps without resting [19]. ADL disability was defined as answering at least one dependency in 7 domains (bathing, continence, dressing, eating, transfer, and washing face and hands). Disability of IADL was defined as answering two or more dependencies in 10 domains (food preparation, household chores, going out for a short distance, grooming, handling finances, laundry, taking personal medication, shopping, using public transportation, and using the telephone) [20]. The Timed Up and Go (TUG) test measured the participants standing up from an armchair of standard height, walking 3 m at their own comfortable and safe gait pace, turn at a marker, return to the chair, and sit down. The TUG time was defined as the time from standing up to sitting down [21]. The Short Physical Performance Battery (SPPB) consists of three standing balance measures (tandem, semitandem, and side-by-side stands), five repeated chair rise tests, and usual gait speed. Each test is scored from 0 to 4 scores, with a total of 12 scores [22]. Nutritional status was assessed using the Korean version of the Mini-Nutritional Assessment Short Form (MNA-SF) [23]. The risk of malnutrition was defined as an MNA-SF score of ≤11 [24].

Comorbidity was defined as ≥2 of the following chronic diseases: hypertension, diabetes, myocardial infarction, peripheral vascular disease, angina, cerebrovascular disease, congestive heart failure, dyslipidemia, rheumatoid arthritis, osteoarthritis, osteoporosis, asthma, or chronic obstructive pulmonary disease [15]. Polypharmacy was defined as taking ≥5 medications [25]. Hearing impairment was defined as the minimum pure-tone average value of >40 dB [26]. Visual impairment was defined as a maximum visual acuity of <0.3 [27]. Blood samples were tested at 8 am after fasting for 8 h.

A participant was determined to be depressed if she/he had a score of ≥6 on the Korean version of the Short Form Geriatric Depression Scale (SGDS-K) [28]. Global cognitive dysfunction was diagnosed if the Korean version of the Mini-Mental State Examination (MMSE-KC) score was <24 [29]. Cognitive impairment was defined as a score of 1.5 standard deviations below the score of the age, sex, and education-matched controls on the cognitive function tests: processing speed (trail making test A), executive function (Frontal Assessment Battery), verbal episodic memory (word list recall test), and working memory (digit span backward) [30]. Quality of life was determined using the EuroQol 5-dimension scale (EQ-5D) [31], EuroQol Visual Analog Scale (EQ-VAS) [32], and 12-items Short Form Health Survey (SF-12) [33]. The SF-12 was used to measure physical and mental health summary [34].

Poor social capital was defined as a lack of participation in social gatherings. Social support was assessed using the Enhancing Recovery in Coronary Artery Disease Social Support Instrument [35,36]. The social network was assessed using the Practitioner Assessment of Network Type Instrument [37]. Interaction with family, friends, and neighbors was dichotomized as high (every day, 2–3/week, or ≥1/week) and low (≤1/month).

### 2.4. Statistical Analysis

We developed age-, sex-, and residence-standardized prevalence. The KFACS population is of nation-wide community-dwelling older adults, but quota sampling stratified by age and sex can limit the generalization of the prevalence rate. To ensure generalization, we performed poststratification adjustment using general population distribution data from the Korean Population and Housing Census conducted by Statistics Korea in 2017. We computed the poststratification adjustments by calibrating the distribution of age (3 groups: 70–74, 75–79, and 80–84 years), sex (2 groups: male and female), and residence (2 groups: urban and rural) in the general population. We calculated mean with standard errors (SE) for continuous variables and frequencies with percentage and 95% confidence intervals (CIs) for categorical variables to investigate the prevalence and characteristics of frailty. We used analysis of variance tests for continuous variables and the chi-square test for categorical variables.

In the unweighted sample, we performed multiple forward stepwise logistic regression analyses to identify the most influential risk factors for frailty. First, we identified the risk factors in each of the 9 domains (sociodemographic, physical, physical function, lifestyle, biological, health condition, medical condition, psychological, and social domain). Then, we identified the risk factors with the strongest association with frailty using the variables selected in the 9 domains. Statistical analyses were performed using SPSS version 25.0 (SPSS Inc., Chicago, IL, USA) and SAS version 9.4 (SAS Institute Inc., Cary, NC, USA). Statistical significance was determined using a two-sided *p*-value of <0.05.

## 3. Results

### 3.1. Sociodemographic Characteristics of the Study Population

The sociodemographic characteristics of the unstandardized and standardized samples are shown in Table 1. The mean age was 75.8 years, and the majority of the participants were aged 70–74 years in both the unweighted (39.7%) and weighted (41.8%) sample populations. There was a significant difference in the regional proportions between men and women in the unweighted sample (*p* = 0.035), but not in the weighted sample (*p* = 0.72).

### 3.2. Prevalence of Frailty

**Figure 1:**
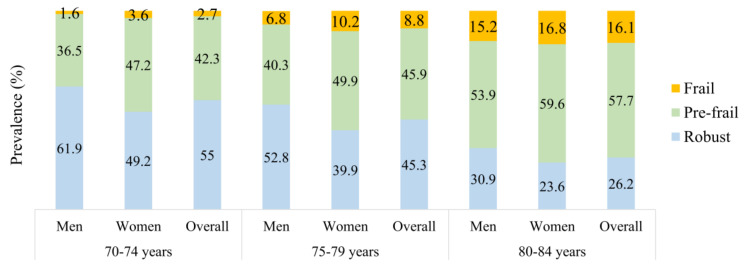
Prevalence of frailty with age groups (standardized sample).

In the standardized sample, the prevalence of frailty and prefrailty was 7.9% (95% CI 6.8–8.9%) and 45.2% (95% CI 45.1–48.8%), respectively. Among the individual frailty components, the prevalence was highest for exhaustion (32.5%), followed by slowness (20.1%) and weakness (19.7%). There was a higher prevalence of exhaustion (40.8% vs. 21.0%) and weakness (21.0% vs. 18.0%) among women compared to men, respectively. However, there was no significant difference in low physical activity, slowness, and unintentional weight loss between women and men. Overall, 54.8% of the participants had ≥1 frailty component (Table 2). The prevalence of frailty increased significantly in the 80–84 years compared to 70–74 years (16.1% vs. 2.7%) (Figure 1). The prevalence of frailty was significantly higher in women than in men in the unstandardized (8.5% and 7.1%) and standardized samples (9.2% and 6.0%) (Table 2 and Table S2). The prevalence of frailty was significantly higher in rural than in cities in the unstandardized (12.0% and 6.2%) and standardized samples (12.7% and 6.0%) (data not shown).

### 3.3. Characteristics of the Study Population across Frailty Status

The characteristics of frailty status in the standardized sample are presented in Table 3. There were significant differences in the sociodemographic (*p* < 0.05), physical (*p* < 0.05), physical function (*p* < 0.001), health condition (*p* < 0.05), and psychological (*p* < 0.001) domains between the three groups. Biological domains, except serum creatinine, cortisol, vitamin B12, thyroid-stimulating hormone (TSH), and low-density lipoprotein (LDL) cholesterol levels were significantly different among the three groups (all, *p* < 0.05). The prevalence of hypertension, diabetes, incontinence, cardiovascular disease, osteoarthritis, osteoporosis, rheumatoid arthritis, digestive system ulceration, and depressive disorder were significantly higher in the frail group (*p* < 0.05). There was a significant difference in lifestyle domain except current smoking (*p* = 0.238) across frailty status. Social domain, except for low interaction with neighbors (*p* = 0.294) and social activities (*p* = 0.491) were also significantly different across frailty status.

### 3.4. Risk Factors Associated with Physical Frailty

**Figure 2:**
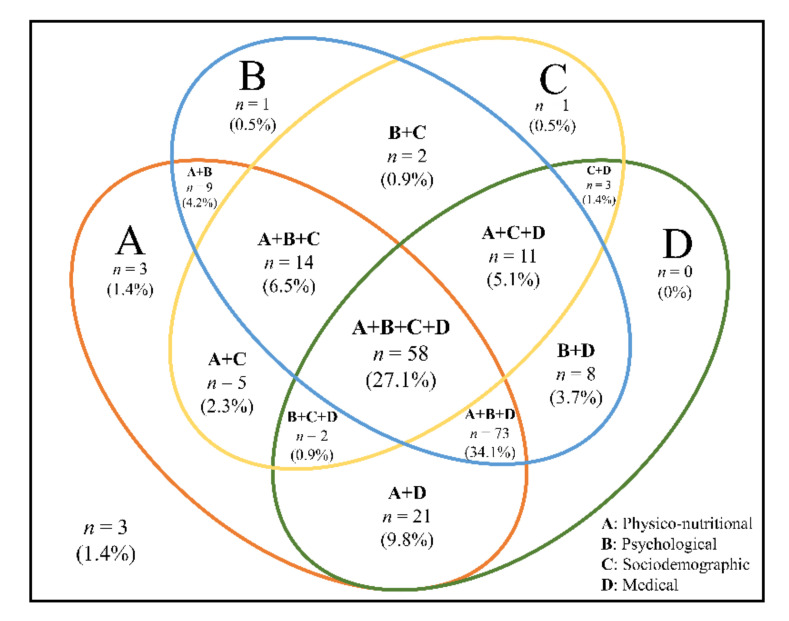
Venn diagram displaying the extent of overlap of risk domains in the frail group (unstandardized sample). A total of 214 adults aged 70–84 years were frail. The physiconutritional domain was defined as having ≥1 risk of malnutrition, sarcopenia, severe mobility limitation, longer Timed Up and Go (>12 s), and low Short Physical Performance Battery (≤9 scores). The psychological domain was defined as having ≥1 depressive symptom and poor self-perceived health. The sociodemographic domain was defined as having ≥1 of rural residence and poor social capital. The medical domain was defined as having ≥1 of polypharmacy, elevated hs-CRP (≥3 mg/L), elevated HbA1c (≥6.5%), and low 25-hydroxyvitamin D (≤20 ng/mL).

Table 4 shows the significant influential risk factors in a multivariate forward logistic regression analysis. Risk factors for frailty were at risk of malnutrition (odds ratio (OR) 2.51; 95% confidence interval (CI) 1.57–4.03), sarcopenia (OR 2.39, 95% CI 1.61–3.56), severe mobility limitation (OR 2.13, 95% CI 1.45–3.15), poor social capital (OR 1.99, 95% CI 1.13–3.56), rural residence (OR 1.89, 95% CI 1.13–3.18), depressive symptoms (OR 1.89, 95% CI 1.29–2.76), poor self–perceived health (OR 1.65, 95% CI 1.12–2.44), polypharmacy (OR 1.61, 95% CI 1.13–2.30), elevated high-sensitivity C-reactive protein (hs-CRP) (OR 1.29, 95% CI 1.07–1.55), elevated glycated hemoglobin (HbA1c) (OR 1.28, 95% CI 1.04–1.56), longer TUG time (OR 1.27, 95% CI 1.17–1.37), and increasing age (OR 1.08, 95% CI 1.03–1.14). High 25-hydroxy vitamin D (OR 0.98, 95% CI 0.96–1.00) and high SPPB scores (OR 0.87, 95% CI 0.76–0.98) were preventable factors. Based on these results, the frequency and percentage of risk factors among frail individuals (n = 214) are shown in Figure 2. For analyses, the significant influential risk factors of frailty presented in Table 4 were classified as physiconutritional, psychological, sociodemographic, and medical domains. About a third (27.1%) of the frail participants had all four risk domains. Overlapping physiconutritional, psychological, and medical risk domains were found in 46.6% of the participants. The prevalence of risk domains in frail participants was as follows: physiconutritional (90.7%), medical (82.2%), psychological (78.0%), and sociodemographic (44.9%) (all, *p* < 0.001) (Figure S1).

## 4. Discussion

Our study was designed to estimate the standardized prevalence of physical frailty using the national standard population composition ratio and to explore comprehensive risk factors for physical frailty among older adults in Korea. Our study showed that the age-, sex-, and residence-standardized prevalence of physical frailty among older adults aged 70–84 years in Korea is 7.9%, increases with age, and is higher among women and those living in rural areas. Furthermore, our study indicates that physiconutritional, medical, psychological, and sociodemographic risk domains were most relevant to physical frailty.

Our study used the FFP to define physical frailty that has been used in many countries and found to predict adverse health outcomes among the older population. In a systematic review, the prevalence of frailty using the FFP varied from 4.0% to 17.0% in community-dwelling older adults aged ≥65 years [3]. The prevalence of physical frailty among Korean community-dwelling adults is comparatively lower than the pooled prevalence of 9.9% (95% CI 9.6–10.2%) in 15 studies [3]. Several studies have estimated the prevalence of frailty using the population structure ratio. Recent epidemiological studies report that the weighted prevalence of frailty using the FFP in community-dwelling older adults varies from 5.2% to 15.2% in Asian countries [11,38,39]. The weighted prevalence of frailty among older adults aged ≥60 years in Singapore was 5.7% (95% CI 4.6–7.1%) and increased significantly with age, with no difference among men and women [11]. In a longitudinal cohort study of a nationally representative sample of community-dwelling adults from 28 provinces in China, the weighted prevalence of frailty was 7.0% and was higher among women than among men (8.0% vs. 5.9%) [39]. This study also observed geographic heterogeneity and urban–rural differences in the prevalence of frailty. In Sri Lankan rural areas, the weighted prevalence of frailty was 15.2% in community-dwelling adults aged ≥60 years, which was higher than that in high- and upper-middle-income countries [38]. The differences in prevalence across countries could be due to the modified components used to define frailty in different studies. The wide variation in the prevalence of frailty has been attributed to the characteristics of a population such as environment, ethnicity, and social culture.

The KFACS recruited participants using quota sampling stratified by age and sex in 10 study centers. To avoid biased results caused by the disproportionate sampling design, adjustment was performed by adjusting for age, sex, and residential areas using the Korean Population and Housing Census conducted by Statistics Korea in 2017. Our study recruited men and women in a 1:1 ratio, with 47.6% men and 52.4% women. However, the proportion of women increased to 57.8% in the standardized sample. These results were consistent with those of previous studies in which the proportion of women increased after age- and sex adjustment [38]. Furthermore, the regional distribution of the overall sample is similar in unstandardized and standardized samples. However, the distribution of residence between men and women was significantly different in the unstandardized sample, but not in the standardized sample. Since the participants were recruited without considering the sex ratio of the residential areas, there may be differences in the residential distribution by sex between unstandardized and standardized samples. The prevalence of physical frailty in the overall samples, in urban and rural areas, was similar regardless of standardization. However, age-, sex-, and residence-adjusted prevalence of frailty was estimated to be lower in men and higher in women than in the unstandardized sample. Similarly, the prevalence of frailty differed after weighting in the community-dwelling aged ≥55 years in Beijing, China [40]. The overall weighted and unweighted prevalence of frailty was estimated to be 9.1% and 12.3%, respectively. Additionally, the prevalence of frailty according to sex and residential area was estimated to be lower after sex and age adjustment.

In this nationwide community-dwelling population of Korean older adults, we found that 7.9% of Korean adults aged 70–84 years were frail. A similar prevalence (7.8%) was reported in Korean community-dwelling older adults aged 65 years and older using the data from the Living Profiles of Older People Survey based on home visits in 2008 [41]. In contrast, the prevalence in our study was lower than that reported in a previous Korean hospital-based study [8]. This could be because our study population (70–84 years) was younger than that in the previous study population involving oldest–old (≥85 years). Moreover, the KFACS participants were ambulatory community-dwelling older adults who may be less frail compared to hospital-based participants. Our study showed that the standardized prevalence of frailty in rural areas was 12.7%, which was lower than that in the Pyeongchang rural area in Korea (12.7% vs. 17.4%). However, the prevalence of prefrailty was similar (52.0% vs. 52.6%) [9]. Both studies recruited ambulatory community-dwelling older adults. The prevalence of frailty may differ depending on the area of residence.

Physical frailty requires a comprehensive range of prevention and management [42], and it is important to identify risk factors for physical frailty in multidimensional domains. We have explored risk factors in a comprehensive range of multidimensional domains. Our study shows that physiconutritional, psychological, sociodemographic, and medical domains are the strongest risk factors for frailty among the 9 domains in older adults. In the physical frail population, participants with all domains (27.1%) were more common than those with none (1.4%) or one (0–1.4%) of the four domains. Our results show that participants have overlapping risk factors and need to manage modifiable risk factors using a multidimensional approach. In the physical domain, we found a correlation between sarcopenia and physical frailty. By definition, sarcopenia includes a low physical function, which means that sarcopenia is an essential component of physical frailty [42]. Previous studies have shown that physical frailty is associated with sarcopenia, and both conditions tend to overlap [43,44]. Therefore, sarcopenia should be considered in the management of physical frailty, as suggested in recent international clinical practice guidelines [42]. In addition, our study identified a significant correlation between physical frailty and physical function domains, including severe mobility limitation, longer TUG times, and lower SPPB scores. We have identified the correlation between physical function and frailty in cross-section, and physical function has been used as a simple tool for physical frailty. In a systematic review, the TUG test was found to have a high sensitivity for identifying physical frailty [45]. SPPB scores might also be used as a screening tool to detect physical frailty and correlate with physical frailty in community-dwelling older adults [46]. These physical functions cannot be used as a single test to diagnose physical frailty, but can help assess physical frailty. Our finding of a strong correlation between physical frailty and polypharmacy is consistent with previous studies [47]. In French older adults aged ≥70 years, polypharmacy with 5–9 drugs (OR 1.77, 95% CI 1.20–2.61) and excessive polypharmacy with 10 drugs or more (OR 4.47, 95% CI 2.37–8.42) were associated with physical frailty. Frail people usually have a number of chronic conditions [1] and may be at risk of polypharmacy. Therefore, physical frailty can be managed by reducing polypharmacy through medication management. Our results show that malnutrition has the strongest association with frailty. This association has also been reported in recent cross-sectional studies [48]. Malnutrition is an important pathogenic factor of frailty [49]. International clinical practice guidelines recommend a broad nutritional assessment as part of an appropriate approach to frailty [42,50,51]. In addition, we report a relationship between a low concentration of 25-hydroxyvitamin D and frailty. Because vitamin D deficiency in older adults increases the risk of adverse outcomes such as osteoporosis and low muscle strength, vitamin D might be associated with frailty [52]. We observed a strong correlation between frailty and biological factors. Previous studies have reported a relationship between inflammatory markers and frailty [53,54,55], which is consistent with our results. Additionally, HbA1c, an indicator of diabetes diagnosis, was associated with frailty in our study. Several studies have shown that older adults with diabetes are more likely to be frail than those without diabetes [56,57]. In the psychological domain, physical frailty has been correlated with depressive symptoms. In a systematic review, people with depression were at increased odds of having physical frailty (OR 4.07, 95% CI 1.93–8.55), while frail people were also at increased odds of having depression (OR = 2.64; 95% CI: 1.59–4.37) [58]. As the symptoms of physical frailty and depression are common among older adults and correlated, appropriate interventions are needed. In the social domain, we demonstrated that social capital is related to frailty. Poor social participation can lead to social isolation and loneliness as well as frailty among older adults [59]. In a recent systematic review, there were correlations between physical frailty and social environments including social networks, social support, social participation, subjective neighborhood experience, and sociodemographic neighborhood characteristics. Among them, neighborhood dimensions and social participation had more consistent results. Thus, the social environment should be considered in the management of physical frailty. Our findings of a strong correlation between frailty and age and residence are consistent with previous studies [11,60]. Systematic reviews have shown that physical frailty is a common age-related syndrome, and most studies have been associated with increasing age and physical frailty [61]. Therefore, we should be able to intervene and manage modifiable risk factors.

Our study has several limitations. Due to the cross-sectional design, a causal relationship between risk factors and frailty cannot be determined. The characteristics of the oldest-old (≥85 years) population were unexplored in this study. Despite these limitations, we standardized the study population by sex, age, and residence based on the Korean Population and Housing Census conducted by Statistics Korea in 2017. Furthermore, we examined a comprehensive range of risk factors for frailty status in a homogeneous population. We determined the strongest risk factors associated with frailty.

## 5. Conclusions

The standardized prevalence of physical frailty increases with age and is higher among women and in rural areas. Furthermore, our study showed that multiple domains, such as physiconutritional, psychological, sociodemographic, and medical domains, are strongly associated with physical frailty. Management of modifiable risk factors might help in multidimensional prevention and intervention to reduce physical frailty among the older population in Korea.

## Figures and Tables

**Table 1:** Sociodemographic characteristics of the unstandardized and standardized study samples.

	Unstandardized Sample, n (%)							Standardized Sample (%)			
Variable	Overall n = 2907		Men n = 1383 (47.6%)		Women n = 1524 (52.4%)		*p*-Value	Overall	Men (42.2%)	Women (57.8%)	*p*-Value
Age (years)											
70–74	1154	(39.7)	505	(36.5)	649	(42.6)	**<0.001**	41.8	45.4	39.2	**<0.001**
75–79	1080	(37.2)	529	(38.3)	551	(36.2)		36.1	35.8	36.3	
80–84	673	(23.2)	349	(25.2)	324	(21.3)		22.1	18.8	24.5	
Low education level (<7 years)	1265	(43.5)	361	(26.1)	904	(31.1)	**<0.001**	45.5	25.5	60.5	**<0.001**
Live alone	659	(22.7)	120	(8.7)	539	(35.4)	**<0.001**	24.3	8.2	36.2	**<0.001**
Marital status (without partner)	948	(32.6)	145	(10.5)	803	(52.7)	**<0.001**	35.2	9.9	53.7	**<0.001**
Residence											
Urban	822	(28.4)	387	(28.1)	435	(28.7)	**0.035**	28.4	29.1	27.9	0.720
Suburban	1250	(43.2)	569	(41.4)	681	(45.0)		43.6	43.5	43.6	
Rural	819	(28.3)	420	(30.5)	399	(26.3)		28.0	27.3	28.4	
Social security recipient	204	(7.0)	86	(6.2)	118	(7.8)	0.058	7.2	6.3	7.9	0.055
Current worker	758	(26.1)	425	(30.8)	333	(21.9)	**<0.001**	25.8	31.1	21.9	**<0.001**

*p* < 0.05 indicated in bold.

**Table 2:** Prevalence of frailty status and component (standardized sample).

	Overall		Men (42.2%)		Women (57.8%)		
Variable	%	(95% CI)	%	(95% CI)	%	(95% CI)	*p*-Value
**Frailty status**							
Robust	45.2	(43.3–47.0)	52.8	(50.1–55.5)	39.6	(37.1–42.0)	**<0.001**
Prefrail	47.0	(45.1–48.8)	41.1	(38.5–43.8)	51.3	(48.7–53.8)	
Frail	7.9	(6.8–8.9)	6.0	(4.9–7.2)	9.2	(7.7–10.7)	
**Frailty component**							
Exhaustion	32.5	(30.7–34.2)	21.1	(18.9–23.2)	40.8	(38.3–43.3)	**<0.001**
Low physical activity	10.2	(8.7–11.8)	10.7	(9.1–12.3)	10.5	(9.4–11.6)	0.695
Slowness	20.1	(18.6–21.6)	18.8	(16.8–20.8)	21.1	(19.0–23.2)	0.131
Weakness	19.7	(18.3–21.2)	18.0	(16.0–20.0)	21.0	(18.9–23.1)	**0.042**
Unintentional weight loss	4.9	(4.1–5.7)	5.2	(4.0–6.3)	4.6	(3.5–5.7)	0.527
**Frailty score**							
0	45.2	(43.3–47.0)	52.8	(50.1–55.5)	39.6	(37.1–42.0)	**<0.001**
1	32.1	(30.3–33.8)	28.8	(26.3–31.2)	34.5	(32.1–36.9)	
2	15.0	(13.6–16.3)	12.4	(10.7–14.1)	16.8	(14.9–18.7)	
3	5.8	(4.9–6.7)	4.5	(3.4–5.5)	6.8	(5.5–8.1)	
4	1.9	(1.4–2.4)	1.5	(1.0–2.1)	2.2	(1.4–2.9)	
5	0.1	(0.0–0.3)	0.1	(0.0–0.2)	0.2	(0.0–0.4)	

CI, confidence interval. *p* < 0.05 indicated in bold.

**Table 3:** Characteristics of the standardized study sample according to frailty status.

Variable	Overall			Robust (45.2%)			Prefrail (47.0%)			Frail (7.9%)			*p*-Value
***Sociodemographic***													
Age (years)	75.8	±	0.07	74.9	±	0.09	76.3	±	0.10	78.5	±	0.23	**<0.001**
70–74	41.8			51.0			37.7			14.5			**<0.001**
75–79	36.1			36.2			35.2			40.4			
80–84	22.1			12.8			27.1			45.2			
Female sex	57.8			50.6			63.1			67.5			**<0.001**
Low education level (<7 years)	45.5			33.7			52.5			71.8			**<0.001**
Live alone	24.3			19.4			27.5			33.6			**<0.001**
Marital status (without partner)	35.3			29.3			39.2			46.2			**<0.001**
Residence													
Urban	28.4			34.2			25.5			13.2			**<0.001**
Suburban	43.6			43.9			43.6			41.7			
Rural	28.0			21.9			30.9			45.2			
Social security recipient	7.2			5.8			8.0			11.1			**0.017**
Current worker	25.8			26.2			26.6			17.9			**0.010**
***Physical***													
Underweight (BMI < 18.5 kg/m^2^)	1.6			1.1			1.7			4.0			**0.030**
Low appendicular skeletal muscle (lower 20%)	22.3			16.3			25.9			35.6			**<0.001**
Low calf circumference (<32 cm)	27.6			19.5			31.9			48.1			**<0.001**
High waist circumference (M ≥ 102 cm; F ≥ 88 cm)	51.4			48.1			54.1			53.6			**0.007**
Sarcopenia (AWGS-defined)	10.1			1.1			14.6			34.5			**<0.001**
ADL disability (>1 point)	2.2			0.8			2.1			10.2			**<0.001**
IADL disability (>2 points)	6.3			3.9			6.8			17.3			**<0.001**
Falls in the past year	20.6			16.0			22.3			36.6			**<0.001**
***Physical function***													
Severe mobility limitation	10.1			1.1			14.6			34.5			**<0.001**
Timed Up and Go (seconds)	10.5	±	0.05	9.4	±	0.04	10.8	±	0.1	14.9	±	0.3	**<0.001**
Short Physical Performance Battery (score)	10.8	±	0.03	11.4	±	0.02	10.6	±	0.04	8.6	±	0.15	**<0.001**
Gait speed (m/s)	1.10	±	0.00	1.22	±	0.01	1.04	±	0.00	0.76	±	0.01	**<0.001**
Grip strength (kg)	25.7	±	0.1	28.7	±	0.2	24.0	±	0.2	18.9	±	0.4	**<0.001**
***Biological***													
Albumin (g/dL)	4.4	±	0.00	4.4	±	0.01	4.3	±	0.01	4.3	±	0.02	**<0.001**
Serum creatinine (mg/dL)	0.84	±	0.01	0.83	±	0.01	0.84	±	0.01	0.87	±	0.02	0.271
HbA1c (%)	6.0	±	0.02	6.0	±	0.02	6.0	±	0.02	6.2	±	0.08	**<0.001**
WBC (X1000/uL)	5.9	±	0.03	5.7	±	0.04	6.0	±	0.05	6.2	±	0.12	**<0.001**
RBC (Mil/uL)	4.4	±	0.01	4.4	±	0.01	4.3	±	0.01	4.2	±	0.03	**<0.001**
Cortisol (ug/dL) at 8 a.m.	10.1	±	0.08	10.2	±	0.10	10.1	±	0.11	10.2	±	0.30	0.740
hs-CRP (mg/L)	1.34	±	0.04	1.29	±	0.05	1.31	±	0.05	1.85	±	0.19	**<0.001**
Vitamin B12 (pg/mL)	610.1	±	5.34	614.8	±	7.77	608.3	±	7.77	593.4	±	22.02	0.551
TSH (ulU/mL)	2.8	±	0.10	2.9	±	0.20	2.6	±	0.07	2.7	±	0.22	0.306
Insulin (uU/mL)	8.0	±	0.18	7.3	±	0.20	8.3	±	0.21	9.9	±	1.41	**<0.001**
Triglyceride (mg/dL)	122.6	±	1.15	120.3	±	1.69	122.5	±	1.60	137.1	±	5.15	**0.001**
Total cholesterol (mg/dL)	174.6	±	0.68	176.5	±	1.01	173.1	±	1.01	172.5	±	2.26	**0.037**
HDL-cholesterol (mg/dL)	52.5	±	0.26	53.4	±	0.39	52.0	±	0.38	50.5	±	0.93	**0.003**
LDL-cholesterol (mg/dL)	108.2	±	0.63	109.7	±	0.95	107.1	±	0.92	106.0	±	2.10	0.069
25-hydroxy vitamin D (mg/mL)	23.2	±	0.19	23.6	±	0.28	23.2	±	0.27	21.0	±	0.56	**0.001**
eGFR (mL/min/1.73 m^2^) ^a^	77.5	±	0.27	78.9	±	0.35	76.9	±	0.41	73.3	±	1.14	**<0.001**
***Lifestyle***													
Current smoker	5.2			4.7			5.3			7.7			0.238
Alcohol intake (≥2–3 time/week)	16.3			19.1			14.1			13.4			**0.001**
Sleep latency (>1 h)	4.3			3.1			4.9			7.0			**0.019**
Long night-time sleep (>8 h)	5.9			4.8			6.5			9.2			**<0.001**
Dairy products (not every day)	60.9			56.3			62.8			74.0			**<0.001**
Legumes and eggs intake (<2 times/week)	19.7			13.8			22.8			33.5			**<0.001**
Meat, fish, and poultry intake (not every day)	80.8			78.0			83.2			82.0			**0.045**
Risk of malnutrition (MNA score ≤ 11)	8.0			3.7			10.0			20.0			**<0.001**
***Health condition***													
Number of drugs	4.4	±	0.06	3.9	±	0.08	4.7	±	0.09	5.7	±	0.23	**<0.001**
Comorbidity (≥2 diseases)	55.6			48.3			61.3			63.2			**<0.001**
Polypharmacy (≥5 medications)	32.2			24.4			36.0			55.0			**<0.001**
Hospitalization in the past year	12.9			8.6			15.8			20.5			**<0.001**
Hearing impairment	15.3			13.9			15.7			21.2			**0.033**
Visual impairment	2.6			1.4			3.4			4.4			**0.002**
Low chewing ability	46.7			39.1			48.8			64.0			**<0.001**
Low pronouncing ability	25.0			18.9			27.4			45.5			**<0.001**
***Medical condition***													
Hypertension	58.4			54.0			61.4			66.2			**<0.001**
Diabetes	21.9			18.3			23.9			31.3			**<0.001**
Urinary incontinence	4.1			2.2			5.0			10.4			**<0.001**
Cardiovascular disease ^b^	13.3			11.2			14.4			18.6			**0.004**
Dyslipidemia	33.6			34.2			33.7			29.1			0.326
Osteoarthritis	26.7			20.1			31.6			34.8			**<0.001**
Osteoporosis	17.5			13.2			20.6			23.4			**<0.001**
Rheumatoid arthritis	2.2			1.0			3.0			4.2			**<0.001**
Digestive system ulceration	6.3			4.9			7.5			7.2			**0.019**
Chronic obstructive pulmonary disease	0.9			0.9			0.8			1.3			0.703
Allergic rhinitis	4.1			4.8			3.6			3.3			0.241
Bronchitis	1.5			1.0			1.9			2.1			0.137
Asthma	3.6			3.1			3.7			5.2			0.369
Thyroid disease	4.7			4.9			4.5			4.4			0.889
Kidney disease	1.5			1.0			1.8			2.8			0.087
Prostate disease	14.7			16.8			13.0			12.6			**0.011**
Depressive disorder	3.0			1.9			2.8			10.7			**<0.001**
***Psychological***													
EQ-5D index	0.88	±	0.00	0.92	±	0.00	0.86	±	0.00	0.73	±	0.01	**<0.001**
EQ-VAS	74.2	±	0.33	79.6	±	0.40	71.3	±	0.49	60.1	±	1.49	**<0.001**
SF-12		±			±			±			±		
Physical health	43.3	±	0.21	48.1	±	0.23	40.9	±	0.30	30.7	±	0.72	**<0.001**
Mental health	52.7	±	0.20	55.6	±	0.22	51.3	±	0.32	44.7	±	0.87	**<0.001**
Poor self-perceived health	31.0			17.2			37.9			68.9			**<0.001**
Depressive symptoms (GDS score ≥ 6)	22.7			9.4			29.8			57.0			**<0.001**
Cognitive dysfunction (MMSE score < 24)	22.3			12.4			27.3			49.6			**<0.001**
Cognitive impairment	24.4			17.9			27.7			41.7			**<0.001**
***Social***													
Social support	5.5	±	0.02	5.5	±	0.03	5.4	±	0.04	5.3	±	0.10	**0.026**
Poor social capital	6.4			5.2			6.3			13.7			**0.002**
Social network													
Low interaction with family	39.5			37.1			40.6			46.4			**0.019**
Low interaction with friends	23.1			17.1			25.8			41.9			**<0.001**
Low interaction with neighbor	28.7			30.2			27.6			26.9			0.294
Religious activities (none)	41.7			41.5			40.6			49.8			**0.044**
Social activities (none)	21.6			20.6			22.3			23.3			0.491

Values are presented as mean ± standard error or percentage. ^a^ eGFR, estimated using the Chronic Kidney Disease Epidemiology Collaboration (CKD-EPI) creatinine equation; M, male; F; female. ^b^ Cardiovascular diseases were included myocardial infarction, congestive heart failure, angina, peripheral vascular disease, and cerebrovascular disease. ADL, activities of daily living; IADL, instrumental activities of daily living; HbA1c, glycosylated hemoglobin; WBC, white blood cell; RBC, red blood cell; hs-CRP high-sensitivity C-reactive protein; TSH, thyroid-stimulating hormone; HDL-cholesterol, high-density lipoprotein; LDL-cholesterol, low-density lipoprotein; eGFR, estimated glomerular filtration rate; MNA, Mini-Nutritional Assessment; EQ-5D, EuroQol-5 dimension; EQ-vas, EuroQol Visual Analogue Scale; SF-12, 12 item short form health survey; GDS, Global Deterioration Scale; MMSE, Mini-Mental State Exam. *p* < 0.05 indicated in bold.

**Table 4:** Risk factors associated with physical frailty in the final forward logistic regression models (unstandardized sample).

Variable	*B*	S.E.	*p*-Value	OR	95% CI	
					Lower	Upper
At risk of malnutrition ^a^	0.922	0.240	<0.001	2.51	1.57	4.03
Sarcopenia ^b^	0.872	0.202	<0.001	2.39	1.61	3.56
Severe mobility limitation ^c^	0.758	0.199	<0.001	2.13	1.45	3.15
Poor social capital ^d^	0.687	0.287	0.017	1.99	1.13	3.49
Residence						
Urban	Ref.					
Suburban	0.571	0.259	0.027	1.77	1.07	2.94
Rural	0.673	0.265	0.016	1.89	1.13	3.18
Depressive ^e^	0.634	0.194	0.001	1.89	1.29	2.76
Poor self-perceived health	0.502	0.199	0.012	1.65	1.12	2.44
Polypharmacy ^f^	0.478	0.181	0.008	1.61	1.13	2.30
hs-CRP, mg/L	0.252	0.095	0.008	1.29	1.07	1.55
HbA1c, %	0.243	0.103	0.018	1.28	1.04	1.56
Timed Up and Go, seconds	0.236	0.039	<0.001	1.27	1.17	1.37
Age, years	0.081	0.025	0.001	1.08	1.03	1.14
25-hydroxyvitamin D, ng/mL	−0.023	0.010	0.024	0.98	0.96	1.00
Short Physical Performance Battery	−0.144	0.064	0.025	0.87	0.76	0.98

Independent forward stepwise logistic regression analysis with adjustment for multiple comparisons. Controlled age, education level, residence, current worker, low calf circumference, sarcopenia, severe mobility limitation, ADL disability, IADL disability, fall in the past year, Timed Up and Go, Short Physical Performance Battery, albumin, serum creatinine, hemoglobin A1c, HbA1c, red blood cell, free thyroxine, triglyceride, 25-hydroxyvitamin D, estimated glomerular filtration rate, risk of malnutrition, polypharmacy, hospitalization in the past year, low pronouncing ability, diabetes, urinary incontinence, osteoarthritis, rheumatoid arthritis, EuroQol-5 dimensions, depressive symptoms, cognitive impairment, social support, poor social capital, low interaction with friends, and social activities. ^a^ At risk of malnutrition: Mini-Nutritional Assessment Short Form score of ≤11. ^b^ Sarcopenia: defined according to the consensus report of the Asian Working Group for sarcopenia. ^c^ Severe mobility limitation: “very difficult” or “impossible” to either walk about 400 m or climb 10 steps without resting. ^d^ Poor social capital: any lack of participation in social gatherings. ^e^ Depressive: a score of ≥6 on the Korean version of the Short Form Geriatric Depression Scale (SGDS-K). ^f^ Polypharmacy: taking ≥5 medications. hs-CRP, high-sensitivity C-reactive protein; HbA1c, glycosylated hemoglobin; *B*, regression coefficient; S.E., standard error; OR, odds ratio; CI, confidence interval.

## Supplementary Materials

The following are available online at https://www.mdpi.com/1660-4601/17/21/7883/s1, Figure S1: The proportion of risk domains across the frailty status (unstandardized sample), Table S1: Criteria used to physical frailty, and Table S2: Prevalence of frailty status and component (unstandardized sample).
