# Supplementary material for: Prevalence of Physical Frailty and Its Multidimensional Risk Factors in Korean Community-Dwelling Older Adults: Findings from Korean Frailty and Aging Cohort Study
Source: Int J Environ Res Public Health. 2020 Oct 27;17(21):7883. doi: 10.3390/ijerph17217883 (PMC7662797; doi:10.3390/ijerph17217883)
Supplement: Supplementary file 1 [file ijerph-17-07883-s001.pdf]

Supplementary materials

Table S1. Criteria used to define physical frailty

	CHS	KFACS
**Weight loss**	In the last year, have you lost more than 10 pounds(4.54kg) unintentionally?<br>□ No □ Yes<br>If answer is yes, this criterion is positive.	Same as in CHS
**Weakness**	Grip strength stratified by gender and BMI quartiles: JAMAR hydraulic hand dynamometer (lowest 20%)<br>Men<br>BMI ≤24.0…≤29.0 kg<br>BMI 24.1–26.0…≤30.0 kg<br>BMI 26.1–28.0…≤30.0 kg<br>BMI >28.0…≤32.0 kg<br>Women<br>BMI ≤23.0…≤17.0 kg<br>BMI 23.1–26.0…≤17.3 kg<br>BMI 26.1–29.0…≤18.0 kg<br>BMI >29.0…≤21.0 kg<br>If grip strength is lower than these respective cut-offs, the criterion is positive	Grip strength stratified by gender and BMI quartiles: Digital Smedley Dynamometer (T.K.K.5401, Takei Scientific Inc. Co., Ltd., Japan) (lowest 20%)<br>Men<br>BMI <22.0…≤25.4 kg<br>BMI 22.0–23.9…≤27.1 kg<br>BMI 24.0–25.9…≤27.8 kg<br>BMI >26.0…≤28.5 kg<br>Women<br>BMI <23.0…≤16.8 kg<br>BMI 23.0–24.9…≤17.6 kg<br>BMI 25.0–26.9…≤17.8 kg<br>BMI >27.0…≤17.7 kg<br>If grip strength is lower than these respective cut-offs, the criterion is positive
**Exhaustion**	Using the CES-D, the following two statements are read asking how often in the last week did you feel this way?<br>• I felt that everything I did was an effort<br>• I could not get going<br><br>0 = rarely or none time (<1 day)<br>1 = some or little time (1–2 days)<br>2 = a moderate amount of the time (3–4days)<br>3 = most of the time<br>Persons answering "2" or "3" to either of these questions are categorized as positive	Same as in CHS
**Slowness**	Gait speed stratified by gender and height (gender-specific cutoff a medium height): 4.57-m usual gait speed (lowest 20%)<br>Men<br>Height ≤173.0 cm …≤ 0.76 m/s<br>Height >173.0 cm …≤ 0.65 m/s<br>Women<br>Height ≤159.0 cm …≤ 0.76 m/s<br>Height >159.0 cm …≤ 0.65 m/s<br>If gait speed is lower than these respective cut-offs, the criterion is positive	Gait speed stratified by gender and height (gender-specific cutoff a medium height): 4-m usual gait speed (lowest 20%)<br>Men<br>Height ≤165.0 cm …≤ 0.93 m/s<br>Height >165.0 cm …≤ 0.98 m/s<br>Women<br>Height ≤152.0 cm …≤ 0.85 m/s<br>Height >152.0 cm…≤ 0.93 m/s<br>If gait speed is lower than these respective cut-offs, the criterion is positive
**Low activity**	Kcal of leisure physical activity stratified by gender: MLTAQ<br>Men <383 Kcal<br>Women <270 Kcal<br>If Kcal of leisure physical are under these respective cut-offs, the criterion is positive	Kcal of self-report of physical activity stratified by gender: IPAQ-SF<br>Population-based Korean survey of older adults (lowest 20%)[a]<br>Men <495.65 Kcal<br>Women <283.50 Kcal<br>If Kcal of leisure physical are under these respective cut-offs, the criterion is positive

BMI, body mass index; CES-D, Center for Epidemiological Studies-Depression Scale; CHS, Cardiovascular Health Study; IPAQ-SF, the International Physical Activity Questionnaire - Short Form; KFACS, Korean Frailty and Aging Cohort Study; MLTAQ, the Minnesota Leisure Time Activities Questionnaire. [a]Ministry of Health and Welfare. Survey on health and welfare status of the elderly in Korea 2008 [Internet]. Seoul: Ministry of Health and Welfare; 2012. cited 2009 Jul 13. Available from: http://www.mw.go.kr/.

Table S2. Prevalence of frailty status and component (unstandardized sample)

Variable	Unstandardized sample, n (%) Overall n=2,907		Men n=1,383 (47.6%)		Women n=1,524 (52.4%)		*p* Value
**Frailty status**							
Robust	1313	(45.2)	695	(50.3)	618	(40.6)	
Pre-frail	1366	(47.0)	590	(42.7)	776	(50.9)	**<0.001**
Frail	228	(7.8)	98	(7.1)	130	(8.5)	
**Frailty component**							
Exhaustion	915	(31.5)	306	(33.4)	609	(66.4)	**<0.001**
Low physical activity	310	(10.7)	153	(11.1)	157	(10.3)	0.507
Slowness	590	(20.3)	284	(20.5)	306	(20.1)	0.760
Weakness	588	(20.0)	281	(20.3)	307	(20.1)	0.907
Unintentional weight loss	141	(4.9)	74	(5.4)	67	(4.4)	0.232
**Frailty score**							
0	1313	(45.2)	695	(50.3)	618	(40.6)	
1	936	(32.2)	404	(29.2)	532	(34.9)	
2	430	(14.8)	186	(13.4)	244	(16.0)	
3	168	(5.8)	71	(5.1)	97	(6.4)	**<0.001**
4	56	(1.9)	26	(1.9)	30	(2.0)	
5	4	(0.1)	1	(0.1)	3	(0.2)	

$p < 0.05$ indicated in bold.

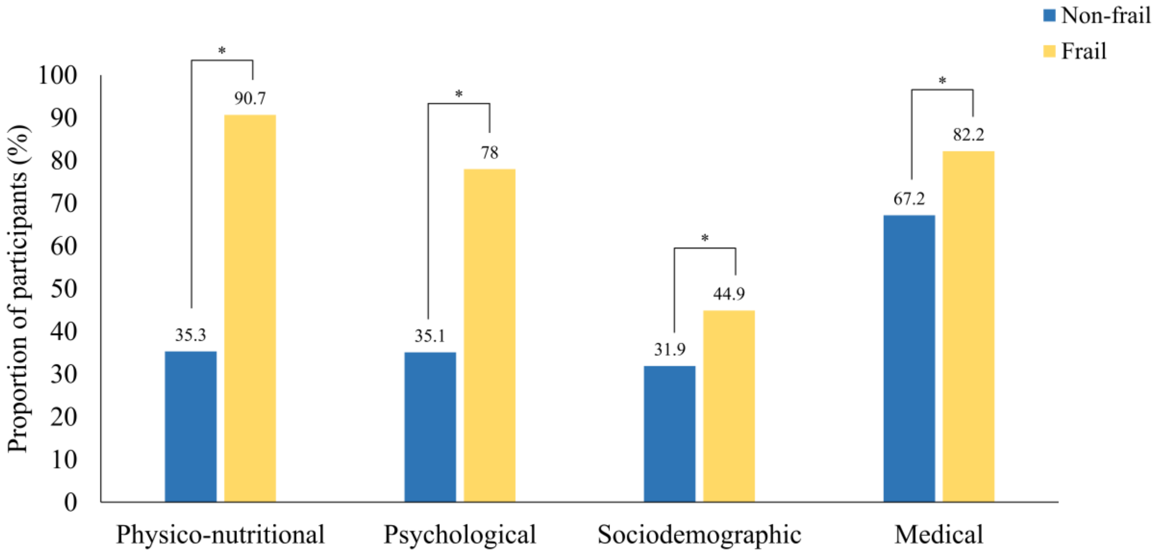

Figure S1. The proportion of risk domains across the frailty status (unstandardized sample); The frailty status was classified into non-frail and frail. The physico-nutritional domain was defined as having ≥1 risk of malnutrition, sarcopenia, severe mobility limitation, longer timed up and go (>12 seconds), and low short physical performance battery (≤9 scores). The psychological domain was defined as having ≥1 depressive symptom and poor self-perceived health. The sociodemographic domain was defined as having ≥1 of rural residence and poor social capital. The medical domain was defined as having ≥1 of polypharmacy, elevated hs-CRP (≥3 mg/L), elevated HbA1c (≥6.5%), and low 25-hydroxyvitamin D (≤20 ng/mL). * $p < 0.001$.
